# Standardization of a screening instrument (PHQ-15) for somatization syndromes in the general population

**DOI:** 10.1186/1471-244X-13-91

**Published:** 2013-03-20

**Authors:** Rüya-Daniela Kocalevent, Andreas Hinz, Elmar Brähler

**Affiliations:** 1 Institute and Policlinic for Medical Psychology, University Medical Center Hamburg-Eppendorf, Martinistr. 52, W26, Hamburg, 20246, Germany; 2 Department of Medical Psychology and Medical Sociology, University of Leipzig, Ph.-Rosenthal-Str. 55, Leipzig, 04103, Germany

**Keywords:** Somatization, Standardized data, PHQ-15, General population

## Abstract

**Background:**

The PHQ-15 is widely used as an open access screening instrument for somatization syndromes in different health care settings, thus far, normative data from the general population are not available. The objectives of the study were to generate normative data and to further investigate the construct validity of the PHQ-15 in the general population.

**Methods:**

Nationally representative face-to face household surveys were conducted in Germany between 2003 and 2008 (n=5,031). The survey questionnaires included, the 15-item somatization module from the Patient Health Questionnaire (PHQ-15), the 9-item depression module (PHQ-9), the Satisfaction With Life Scale (SWLS), the SF-12 for the measurement of health related quality of life, and demographic characteristics.

**Results:**

Normative data for the PHQ-15 were generated for both genders and different age levels including 5031 subjects (53.6% female) with a mean age (SD) of 48.9 (18.1) years. Somatization syndromes occured in 9.3% of the general population. Women had significantly higher mean (SD) scores compared with men [4.3 (4.1) vs. 3.4 (4.0)]. Intercorrelations with somatization were highest with depression, followed by the physical component summary scale of health related quality of life.

**Conclusions:**

The normative data provide a framework for the interpretation and comparisons of somatization syndromes with other populations. Evidence supports reliability and validity of the PHQ-15 as a measure of somatization syndromes in the general population.

## Background

Somatization is one of the most common issues in health care services, associated with substantial functional impairment and health care utilization [1-3]. Their valid and reliable acquisition is urgently necessary. Somatoform symptoms often account for sick leave and are characterized by long duration and medically unexplained symptoms [4-7]. The most frequently reported symptoms are fatigue, low energy, sleeping trouble, and pain (back pain, headaches, abdominal pain, and chest pain) [8,9]. Medically unexplained symptoms are one of the key features of somatoform disorders. Although they are currently treated as both categorical (in terms of the diagnosis of somatoform disorders) and dimensional (in terms of quantitative measures of somatization/somatic symptom reporting), little is known about the empirical latent structure of medically unexplained symptoms. Accordingly to recent study results, the latent structure of somatization/somatic symptom reporting as assessed by the PHQ-15 is dimensional in both primary care and student samples [10].

Estimated prevalence rates of undifferentiated somatoform disorders vary between 8.6%-25.6% in primary care, depending on the chosen screening instrument and whether pain is taken into account or not [8,11-13]. Recent reported data on somatoform symptom clusters in the general population are still scarce [14]. Wittchen and colleagues (2011) reported in their systematic review a 12-month prevalence of somatoform disorders of 6.3% in the EU with little evidence for considerable cultural or country variation [15]. 4-week, 12-month and lifetime prevalence rates of any somatoform disorder in the German general population was reported with 7.5%, 11.0%, and respectively 16.2% [16].

Most clinicians nowadays evaluate, whether or not the reported somatoform complaints are associated with distress and psychological impairment, both predictors for somatoform disorders [17]. Screening instruments can add valuable diagnostic information, yet they vary considerably in length and diagnostic focus (for an overview of measures used in clinical trials of somatoform disorders see [18]). Patients often complain about the amount of items, which can lead to a difficult doctor-patient relationship and lower self-perception of quality of life and life satisfaction [1,19-21]. The reported impairment of every day functioning can be even higher when the patients are affected by comorbid conditions as depression and/or anxiety, which occurs in up to 43% with increasing number of physical symptoms [11,17,22]. A difficult encounter, as perceived by the clinician, may be another predictor of psychiatric comorbidity in patients who have somatoform symptoms [22]. The collaboration between patients and their doctors might also carry the risk of shaping, reinforcing, and legitimizing somatoform syndromes [23]. Hence it is important to take standardized assessment of somatization into account. These measures can have a variety of uses, including screening, early pre-diagnosis, assessment of severity, and gauging treatment decisions of both clinicians and patients.

The PHQ-15 is a self-administered somatic symptoms subscale, derived from the full Patient-Health-Questionnaire [7,24]. Relatively brief, it screens for 15 somatic symptoms that account for more than 90% of the physical complaints reported in the outpatient setting (exclusive of self-limited upper respiratory symptoms) [20]. The PHQ-15 is a valid measure, which has been used in 40 studies so far in different health care settings (for an overview see [11]). Valid and reliable measures for the assessment of somatic symptoms, as the PHQ-15, have been used in psychiatric research and routinely in clinical practice so far (i.e. primary care). Normative data, which could be used to compare a subject's scale score with those determined from a general population reference group, are still scarce and restraint to relative risk factors [25]. The obtained findings could be further utilized as reference categories in community studies and open-access web-based screening tools [15,26,27].

In this study we provide normative data for the PHQ-15 for different age groups and both genders. In addition we address the relations of somatic symptoms with depression, and quality of life and life satisfaction to provide further evidence for the construct validity in a general population. According to previous results, we expect that higher PHQ-15 scores will be associated with worsening quality of life and life satisfaction as well as with increased depression [11].

## Methods

### Study sample

Nationally representative face-to face household surveys were conducted in Germany between 2003 and 2008 (n=5,031), representative of the German general population, with the assistance of an institute specialized for demographic research (USUMA, Berlin) according to the German law of data protection (§30a BDSG) and with written consent. Previously ethics were weighted to the respective interests of the public and of the individuals concerned following §823 (BGB) of the Civil Code of Law and in accordance with the guidelines in the Declaration of Helsinki. Representativeness was assured through a weighting process. Age, gender, and educational level were the major criteria for representativeness according to the register of the Federal Elections. Two callbacks had to be without success before an address was considered a failure. The sampling procedure consisted of sample points, household, and persons in the last stage. Target households within the sample points were determined using the random-route procedure: choosing sample point areas within Germany, randomly choosing households within these areas, and randomly choosing target persons within these households.

### Sample characteristics

Attempts were made to contact 8008 persons. The set of questionnaires was administered to a sample of 5031 persons. Therefore the response rate was 62.8%. The main reasons for non-participation (37.2%) were: the general information request was refused (15.8%), the interview request was refused (7.9%), or there was no one at home for three times in a row (7.3%).

Sociodemographic characteristics of the sample are reported in Table 1. The analysis of the distribution of the data yielded skewness and kurtosis values of somatization of 1.63 and 3.29, respectively. We therefore decided to investigate group differences for sociodemographic characteristics using non-parametric tests.

**Table 1:** Demographic characteristics of the study sample and associations with PHQ-15 scores (N=5,031)

	**N (%)**	**PHQ-15 score M (SD)**	**Group differences**^**a **^**p-value**	**Cohen’s *d*, effect-size**
Gender			χ^2^(28)=144.5; p<0.001	*d*=0.22
Male	2332 (46.4)	3.4 (4.0)		
Female	2699 (53.6)	4.3 (4.1)		
Age group, yr			χ^2^(6)=425.1; p<0.001	*d*=0.91
14-24	564 (11.2)	2.4 (3.5)		
25-34	630 (12.5)	2.8 (3.5)		
35-44	938 (18.6)	3.2 (3.7)		
45-54	871 (17.3)	3.6 (3.8)		
55-64	844 (16.8)	4.5 (4.1)		
65-74	792 (15.7)	4.8 (4.2)		
≥ 75	392 (7.8)	6.3 (4.9)		
Living with a partner			χ^2^(28)=34.5; p=0.75	-
Yes	2927 (58.3)	3.8 (3.9)		
No	2092 (41.7)	4.0 (4.2)		
Education			χ^2^(4)=123.3; p<0.001	*d*=0.74
None	91 (1.8)	5.1 (4.5)		
Highschool	3878 (77.3)	3.9 (3.9)		
College	525 (10.5)	3.7 (4.0)		
University	344 (6.9)	3.3 (3.3)		
Currently student	181 (3.6)	2.2 (3.2)		
Unemployment				
Yes	297 (5.9)	4.3 (4.2)	χ^2^(28)=52.4; p=0.003	*d*=0.12
No	4722 (94.1)	3.8 (4.1)		
Net household income^b^				
<1250 € / mo	1301 (27.3)	4.8 (4.4)	χ^2^(2)=143.2; p<0.001	*d*=0.45
1250-<2500 € / mo	2499 (52.5)	3.7 (3.9)		
≥ 2500 € / mo	964 (20.2)	2.9 (4.0)		

^a^ Group differences were performed using χ^2^-test and Kruskal-Wallis-test (p<0.001).

^b^ USD$1=EUR€0.77.

There were significant gender, age, education level, employment status, and income effects in the general population associated with a higher PHQ-15 score. The most marked group and the lowest groups were considered calculating the value of Cohen’s *d* using the means and standard deviations. As noted in Table 1, the calculated effect sizes were moderate for income and education, and high for age. Gender and employment status yielded small effect sizes.

### Instruments

#### Somatization (PHQ-15)

Somatization was measured using the somatic symptom module of the PHQ, the PHQ-15 [7,28]. The items include the most prevalent DSM-IV somatization disorder somatic symptoms [29]. Subjects were asked for the last 4 weeks to rate the severity of 13 symptoms as 0 (“not bothered at all”), 1 (“bothered a little”), or 2 (“bothered a lot”). Two additional physical symptoms - feeling tired or having little energy, and trouble sleeping – are contained in the PHQ-9 depression module. For scoring, response options for these two symptoms are coded as 0 (“not at all”), 1 (“several days”), or 2 (“more than half the days” or “nearly every day”).

Thus, the total PHQ-15 score ranges from 0 to 30 and scores of ≥5, ≥10, ≥15 represent mild, moderate and severe levels of somatization. The reliability and validity of the PHQ-15 are high in clinical and occupational health care settings [2,7,11].

#### Depression (PHQ-9)

Depression was assessed with the PHQ nine item depression module (PHQ-9) [30]. Each of the nine PHQ depression items corresponds to one of the DSM-IV Diagnostic Criterion A symptoms for major depressive disorder [29]. Subjects were asked how often, over the last two weeks, they have been bothered by each of the depressive symptoms. Response options are “not at all”, “several days”, “more than half the days”, and “nearly every day”, scored as 0, 1, 2 and 3, respectively. PHQ-9 scores range from 0 to 27, with scores of ≥5, ≥10, ≥15, representing mild, moderate and severe levels of depression severity [31]. Psychometric properties of the PHQ-9 are well documented (for an overview see [11]).

#### Quality of life (SF-12)

The SF-12 is an ubiquitary adopted generic questionnaire on the subjectively perceived health-related quality of life and records the overall subjective state of health of adults for different diseases, in relation to their physical, psychological, and social aspects [32]. A longer version of the SF-12, the SF-20, was already previously used to assess functional impairment in combination with the PHQ-15 [7,17].

SF-12 scales are namely: general health, physical functioning, role physical, bodily pain, vitality, social functioning, mental health, role emotional, yielding the summary scales physical- and mental health.

#### Life satisfaction (SWLS)

Satisfaction with life was measured with the Satisfaction With Life Scale, designed to measure global cognitive judgments of satisfaction with one's life, and consists of five items [33,34]: “In most ways my life is close to my ideal”, “The conditions of my life are excellent”, “I am satisfied with my life”, “So far I have gotten the important things I want in life”, and “If I could live my life over, I would change almost nothing”.

Respondents indicated the extent to which they agreed with each item on a seven-point Likert scale ranging from “strongly agree” to “strongly disagree”. Translations of the SWLS into various languages are available and psychometric properties have been reviewed [35].

#### Internal consistencies

The parameter of internal consistency (Cronbach’s α) for the PHQ15 scale reached the value of α =0.82, for the PHQ-9 α=0.88 respectively. The Satisfaction with Life Scale showed a very good Cronbach’s α of 0.91. Cronbach’s α for the mental component scale (MCS) was 0.84, 0.91 respectively for the physical component scale (PCS) of the SF-12.

### Data analysis

For reliability, internal consistency of the PHQ-15 was assessed. Base rates for single symptoms were calculated using frequency analysis. Descriptive statistics included analyses of prevalence. To determine prevalence rates, a cut-off score of ≥10 was used on the PHQ-15 because the range of ≥10 up to 30 reflects medium and high somatic symptom severity, respectively [7]. The selection of this cut-off score resulted in previous studies in a sensitivity of 80.2% und specifity of 58.5% for a somatoform disorder [3]. For construct validity, we investigated PHQ-15 scale intercorrelations with the PHQ-9 [7,30], the SF-12 [32], and the Satisfaction With Life Scale [33]. In addition, we investigated group differences for sociodemographic characteristics using *χ*^*2*^*-test* and Kruskal-Wallis-test, respectively. Based on results from previous studies with the PHQ-15, we expected that women would have higher somatization scores compared with men and that levels of somatization increase with age and lower levels of education [8]. To provide normative data for the PHQ-15, we generated age- and gender specific percentiles for the PHQ-15 total score. Sample size was sufficient to be divided into gender-specific age groups comprising 10 years each. Statistical analyses were conducted using SPSS with an α-level of 1%. According to previous other studies with the PHQ-15 [11,17], missing values were replaced with the mean value of the remaining items if the number of missing items was below 20%. If the number of missing items in the scale exceeded 20%, the sum score was not computed and counted as missing.

## Results

### Prevalence of somatization syndromes

By using the cut-off scores described below, the total prevalence of somatization syndromes at a moderate to high level was estimated to be 9.3%; 8.1% of the men and 10.3% of women had a PHQ-15 sum score ≥10.

### Base rates of single symptoms

**Figure 1:**
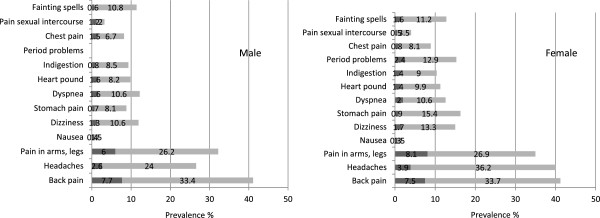
**Gender-stratified base rates of somatoform symptoms.** Symptoms for which the subject had been “bothered a lot” are indicated by the black part of the bar and defined as severe.

The gender-stratified prevalence rates of the individual symptoms are shown in Figure 1. The most common symptoms were various types of pain (back pain, headache, pain of the joints and extremities) with prevalence rates >35%, if symptom reporting of any degree of severity was considered for both genders. Highest rates for severe symptom rating were found for the same symptoms (>4%). Further 2.4% of the total sample complained about sleeping trouble and 1.4% of a lack of energy nearly every day.

### Construct validity

The intercorrelations between the PHQ-15 total score and the PHQ-9 depression scale, the SF-12 for the assessment of quality of life (physical and mental factor), and the Satisfaction with Life Scale are summarized in Table 2. Intercorrelations with somatization were highest with depression (*r*=0.75 p<0.001), followed by the physical component summary scale of health related quality of life (*r*=−0.64 p<0.001), and the subscale “bodily pain” respectively (*r*=−0.68 p<0,001). Intercorrelation of depression was higher with the mental component summary scale of health related quality of life (*r*=−0.68 p<0.001) than the physical component (*r*=−0.48 p<0,001) compared to somatization. Two items, “feeling tired or having little energy” and “trouble falling asleep, or sleeping too much” represent shared questions between the PHQ-9 and the PHQ-15. Omitted from the somatization scale, the intercorrelation reduced from 0.75 to 0.65 (p<0.001).

**Table 2:** Intercorrelations of somatization, depression, life satisfaction, and health related quality of life (N=5,031)

	** Somatization (PHQ-15)**	** Depression (PHQ-9)**
Depression (PHQ-9)	.75**^a^	**-**
Life satisfaction (SWLS)	-.37**	-.42**
Health related quality of life (SF-12)		
Physical functioning	-.61**	-.50**
Role physical	-.66**	-.58**
Bodily Pain	-.68**	-.57**
General health	-.62**	-.52**
Vitality	-.53**	-.52**
Social functioning	-.56**	-.60**
Role Emotional	-.58**	-.59**
Mental health	-.55**	-.61**
Physical component summary scale	-.64**	-.48**
Mental component summary scale	-.59**	-.68**

^a^ ** p<0.001.

Both somatization and depression were significantly related to life satisfaction.

The associations of the PHQ-15 scores with demographic characteristics are shown in Table 1. As hypothezised, PHQ-15 scores increased with age, and women exhibited higher scores than men. Also in accordance with the hypotheses, scores for somatization syndromes were higher in subjects with lower educational levels compared to subjects with higher educational levels. No differences were found in terms of relationship or employment status.

### Normative data

Table 3 summarizes the normative data for the different age levels and both genders. Percentiles from this table can be used to compare an individual subject’s PHQ-15 score with those determined from the general population reference group based on age and gender.

**Table 3:** Normative data from the general population for the PHQ-15

	**Total**	**Men**							**Women**						
	**14-92 yr**	**14-24**	**25-34**	**35-44**	**45-54**	**55-64**	**65-74**	**≥75**	**14-24**	**25-34**	**35-44**	**45-54**	**55-64**	**65-74**	**≥75**
	**N=5,031**	**N=292**	**N=279**	**N=396**	**N=414**	**N=398**	**N=397**	**N=156**	**N=272**	**N=351**	**N=542**	**N=457**	**N=446**	**N=395**	**N=236**
**M**	3.8	1.8	2.0	2.6	3.3	4.3	4.4	5.8	3.2	3.5	3.7	3.8	4.7	5.2	6.5
**SD**	4.1	3.0	2.8	3.6	3.7	4.1	4.3	5.2	3.8	3.7	3.8	3.8	4.1	4.1	4.7
**Sum**	**Percentile**^**a**^														
**Score**															
0	11.1	0.3	0.4	0.3	0.2	0.3	0.3	0.6	0.4	0.3	0.2	0.2	0.3	0.3	0.4
1	28.7	51.0	38.6	31.2	27.1	16.9	17.7	14.1	30.9	23.9	19.8	19.5	10.1	18.8	4.3
2	41.3	67.5	58.8	47.5	41.2	28.0	30.3	23.1	48.5	36.2	32.9	33.7	18.7	29.2	10.2
3	52.5	77.4	73.6	64.0	53.8	41.2	40.4	32.1	56.6	51.3	48.1	46.8	28.8	39.1	18.7
4	62.1	83.6	81.9	74.6	63.2	52.5	51.5	42.3	67.3	60.4	58.4	57.5	39.2	50.5	29.4
5	70.8	88.0	88.8	84.0	72.6	61.9	59.6	48.7	73.2	68.7	69.9	66.7	50.7	60.7	39.6
6	77.8	90.8	93.5	89.3	80.9	71.5	70.2	56.4	80.1	79.5	78.0	74.0	60.9	70.8	50.2
7	82.4	92.8	94.2	91.9	85.5	78.3	78.0	62.2	84.6	86.0	83.4	80.7	69.7	75.9	60.9
8	85.9	93.2	94.6	92.6	88.4	81.1	80.8	66.7	86.4	89.7	86.9	85.3	75.9	79.7	68.1
9	89.2	94.2	96.4	93.9	90.1	86.1	83.8	74.4	90.8	92.6	90.0	90.2	81.2	84.5	75.7
10	91.7	96.9	97.1	95.4	93.5	89.9	87.6	80.1	93.8	93.4	92.6	92.3	85.3	89.3	79.6
11	93.4	96.9	98.9	96.4	94.9	93.2	89.4	82.7	94.1	94.9	94.3	93.7	88.6	91.4	83.4
12	94.7	97.6	98.9	96.4	96.1	94.2	92.4	86.5	96.0	96.6	95.6	95.4	90.5	93.1	85.1
13	95.9	98.3	98.9	96.7	97.1	95.5	93.6	89.7	96.7	97.2	96.3	96.5	92.7	95.7	88.5
14	97.0	99.3	98.9	97.7	98.1	96.7	95.7	92.3	97.4	97.7	97.6	98.0	94.4	97.0	90.6
15	97.7	99.7	98.9	98.0	98.5	97.2	96.5	94.9	98.2	97.7	98.7	98.5	96.0	98.2	92.8
16	98.2	99.9	98.9	98.7	98.8	98.2	97.0	94.9	98.9	98.3	98.9	98.7	97.1	98.2	95.3
17	98.6	99.9	99.3	98.7	99.0	98.2	97.7	94.9	98.9	98.6	99.1	98.7	98.1	99.5	96.2
18	98.9	99.9	99.6	99.2	99.3	98.5	98.7	95.5	98.9	98.6	99.1	99.1	98.5	99.7	97.0
19	99.2	99.9	99.6	99.2	99.5	98.7	98.7	95.5	99.6	99.4	99.4	99.3	98.5	99.9	97.4
20	99.5	99.9	99.9	99.2	99.5	99.2	98.7	96.2	99.9	99.7	99.4	99.3	99.3	99.9	98.3
21	99.7	99.9	99.9	99.2	99.8	99.9	99.7	98.1	99.9	99.7	96.6	99.3	99.3	99.9	98.7
22	99.8	99.9	99.9	99.2	99.8	99.9	99.7	98.7	99.9	99.7	99.8	99.3	99.3	99.9	98.7
23	99.8	99.9	99.9	99.2	99.8	99.9	99.9	98.7	99.9	99.7	99.8	99.3	99.3	99.9	99.6
24	99.9	99.9	99.9	99.2	99.9	99.9	99.9	99.9	99.9	99.7	99.8	99.3	99.3	99.9	99.9
25	99.9	99.9	99.9	99.7	99.9	99.9	99.9	99.9	99.9	99.7	99.8	99.3	99.9	99.9	99.9
26	99.9	99.9	99.9	99.7	99.9	99.9	99.9	99.9	99.9	99.9	99.8	99.9	99.9	99.9	99.9
27	99.9	99.9	99.9	99.7	99.9	99.9	99.9	99.9	99.9	99.9	99.9	99.9	99.9	99.9	99.9
28	99.9	99.9	99.9	99.9	99.9	99.9	99.9	99.9	99.9	99.9	99.9	99.9	99.9	99.9	99.9
29	99.9	99.9	99.9	99.9	99.9	99.9	99.9	99.9	99.9	99.9	99.9	99.9	99.9	99.9	99.9
30	99.9	99.9	99.9	99.9	99.9	99.9	99.9	99.9	99.9	99.9	99.9	99.9	99.9	99.9	99.9

^a^ Percentiles indicate the rank of the subject compared to other subjects of the same age group and gender.

For example, a PHQ-15 score of 11 in a 30-year-old man indicates a percentile rank of 93.4% in the total population and of 98.9% in a group of subjects of the same age and gender. Likewise, a PHQ-15 score of 11 in a 30-year-old woman corresponds to a percentile rank of 93.4% in the total population and of 94.9% in the same age and gender group.

## Discussion

A main result of this study was the standardization of the PHQ-15 with the provision of normative data from the general population. Given that age and gender specific comparative data were generated based on subgroups consisting of n=156 to 542 subjects each, the sample sizes were sufficient to provide normative data. Results of a standardization study of the Patient Health Questionnaire-4 (PHQ-4) on depression and anxiety, yielded that the German general population could be considered comparable to the American general population [36]. The prevalence rate of 9.3% for somatization syndromes corresponds to previous results of surveys in the general population reporting on any somatoform disorder [16] and can be considered for further exploration for the presence of the spectrum of subclinical to full somatoform disorder in clinical practice [3,7]. Previous studies in the general population on base rates for somatoform symptoms report similar frequencies and dominance of various types of pain [9,14]. In primary care the different pain symptoms are also the most prominent ones, accompanied by “lack of energy” and “trouble sleeping” as an indicator of exacerbation [8].

The present study, including more than 5000 subjects, gives evidence that the PHQ-15 is not only a reliable and valid self-report measure for somatization in health care settings but also in the general population. Specifically, the intercorrelations of the PHQ-15 with the PHQ-9 depression scale (*r* = 0.65-0.75), the SF-12 quality of life scale (*r* = −0.53-0.68), and the life satisfaction scale (*r* = −0.37) are similar to intercorrelations between these concepts in other studies suggesting further construct validity of the PHQ-15 [11]. In the original PHQ-15 validation study, which comprised of 6,000 unselected primary care patients, higher PHQ-15 scores were also strongly associated with worsening function on all six SF-20 scales - a longer version of the SF-12 used in the present study -, as well as increased disability days and health care utilization [7,17]. The impact on the physical component scale of the SF-12 was higher for somatization than for depression. The expressed mental component scale showed higher associations with depression than somatization. The high association of somatization and depression in the present study might be partly explained by the overlap of two items in the PHQ-15 and PHQ-9 (“lack of energy”, “sleep disturbance”). Yet these results of concurrent validity are supported by a former study of the PHQ-15 in relation to depression and general mental health [37]. The comorbidity of somatic, anxiety and depressive symptoms (the “SAD” triad) is well-established [11,20]. Still the concordance could not be found in immunological parameters, where results suggest different immune alterations in somatization syndrome and depression [38]. What is known, is that physiological activity (i.e. heart rate, tension) is high in patients with somatization and may interact with psychological processes [39].

The controverse discussion on the classifying of somatoform disorders, respectively syndromes, would have gone beyond the purpose of this study (for an overview see [18]). Although the PHQ-15 does not explicitly ask for “medically unexplained symptoms”, it is highly associated with clinician-rated somatoform disorder symptom counts [40].

Yet a potential limitation of this general population study is that it did not include standard criterion interviews, which would have allowed for calculating specificity and sensitivity for optimal cut point and construction of a receiver operating characteristic (ROC). The sensitivity and specificity of the PHQ-15, as measured by the concordance with the SCID-I diagnosis of somatoform disorders, has previously been established as 78% and 71%, respectively, in primary care [41]. Another limitation might be that normative data were not reported according to the socioeconomic status.

Reviews have identified effective behavioural and pharmacological interventions for somatoform disorders [42-45], and guidelines are close to be published (e.g. S3-guideline). Reported “green flags” or prognostic factors are so far: (a) proactive coping strategies of the patient, e.g. optimism, motivation for psychotherapy; (b) healthy lifestyle, e.g. balanced diet, relaxation, exercising, and enough sleep; (c) social support; and (d) a good doctor-patient relationship with shared decision making.

Reducing the burdens and enhancing early detection of mental disorders in general requires major shifts in research, clinical practice, and public health by incorporating multidisciplinary models of intervention. The good news is that such changes are under way, as reflected, for example by the experts drafting Research Roadmaps (see http://www.roamer-mh.org) for the European Union and the U.S. (see http://www.nihpromis.org).

## Conclusions

Somatization is one of the most common issues in health care services, associated with substantial functional impairment and health care utilization. Somatization syndromes occur in 9.3% of the general population. Thus validate acquisition of somatoform symptoms is necessary in several health care settings. The PHQ-15 is a good basis for this task. Normative data for the PHQ-15 in the general population were generated for both genders and different age levels and can be used for the interpretation and comparisons with other populations.

## Competing interests

The authors declare that they have no competing interests.

## Authors’ contributions

RK participated in the study design, performed statistical analysis and drafted the manuscript. AH participated in the study design and advised for analysis. EB participated in the sequence alignment and acquisition of data and conceived of the study, and participated in its design and coordination. All authors read and approved the final manuscript.

## Pre-publication history

The pre-publication history for this paper can be accessed here:

http://www.biomedcentral.com/1471-244X/13/91/prepub

## References

[B1] Steinbrecher N Koerber S Frieser D The prevalence of medically unexplained symptoms in primary care Psychosom 2011 52 263 71 10.1016/j.psym.2011.01.007 21565598

[B2] De Vroege L Hoedeman R Nuyen J Validation of the PHQ-15 for somatoform disorder in the occupational health care setting J Occup Rehabil 2011 22 51 58 21785907 10.1007/s10926-011-9320-6 PMC3274689

[B3] Korber S Frieser D Steinbrecher N Classification characteristics of the Patient Health Questionnaire-15 for screening somatoform disorders in a primary care setting J Psychosom Res 2011 71 142 7 10.1016/j.jpsychores.2011.01.006 21843748

[B4] Burton C Weller D Marsden W A primary care symptoms clinic for patients with medically unexplained symptoms: pilot randomised trial BMJ Open 2012 2 e000513 10.1136/bmjopen-2011-000513 PMC3330253 22327629

[B5] Hiller W Fichter MM Rief W A controlled treatment study of somatoform disorders including analysis of healthcare utilization and cost-effectiveness J Psychosom Res 2003 54 369 80 10.1016/S0022-3999(02)00397-5 12670616

[B6] Hiller W Fichter MM High utilizers of medical care: a crucial subgroup among somatizing patients J Psychosom Res 2004 56 437 43 10.1016/S0022-3999(03)00628-7 15094029

[B7] Kroenke K Spitzer RL Williams JB The PHQ-15: validity of a new measure for evaluating the severity of somatic symptoms Psychosom Med 2002 64 258 66 11914441 10.1097/00006842-200203000-00008

[B8] Hanel G Henningsen P Herzog W Depression, anxiety, and somatoform disorders: vague or distinct categories in primary care? Results from a large cross-sectional study J Psychosom Res 2009 67 189 97 10.1016/j.jpsychores.2009.04.013 19686874

[B9] Hiller W Rief W Brahler E Somatization in the population: from mild bodily misperceptions to disabling symptoms Soc Psychiatry Psychiatr Epidemiol 2006 41 704 12 10.1007/s00127-006-0082-y 16794766

[B10] Jasper F Hiller W Rist F Somatic symptom reporting has a dimensional latent structure: results from taxometric analyses J Abnorm Psychol 2012 121 725 38 22642842 10.1037/a0028407

[B11] Kroenke K Spitzer RL Williams JB The Patient Health Questionnaire somatic, anxiety, and depressive symptom scales: a systematic review Gen Hosp Psychiatry 2010 32 345 59 10.1016/j.genhosppsych.2010.03.006 20633738

[B12] Mergl R Seidscheck I Allgaier AK Depressive, anxiety, and somatoform disorders in primary care: prevalence and recognition Depress Anxiety 2007 24 185 95 10.1002/da.20192 16900465

[B13] Barkow K Heun R Ustun TB Identification of somatic and anxiety symptoms which contribute to the detection of depression in primary health care Eur Psychiatry 2004 19 250 7 10.1016/j.eurpsy.2004.04.015 15276656

[B14] Rief W Hessel A Braehler E Somatization symptoms and hypochondriacal features in the general population Psychosom Med 2001 63 595 602 11485113 10.1097/00006842-200107000-00012

[B15] Wittchen HU Jacobi F Rehm J The size and burden of mental disorders and other disorders of the brain in Europe 2010 Eur Neuropsychopharmacol 2011 21 655 79 10.1016/j.euroneuro.2011.07.018 21896369

[B16] Jacobi F Wittchen HU Holting C Prevalence, co-morbidity and correlates of mental disorders in the general population: results from the German Health interview and examination Survey (GHS) Psychol Med 2004 34 597 611 10.1017/S0033291703001399 15099415

[B17] Lowe B Spitzer RL Williams JB Depression, anxiety and somatization in primary care: syndrome overlap and functional impairment Gen Hosp Psychiatry 2008 30 191 9 10.1016/j.genhosppsych.2008.01.001 18433651

[B18] Kroenke K Somatoform disorders and recent diagnostic controversies Psychiatr Clin North Am 2007 30 593 619 10.1016/j.psc.2007.08.002 17938036

[B19] Schumacher S Rief W Brahler E Disagreement in doctor's and patient's rating about medically unexplained symptoms and health care use Int J Behav Med 2011 2011 2011 10.1007/s12529-011-9213-2 22187202

[B20] Kroenke K Patients presenting with somatic complaints: epidemiology, psychiatric comorbidity and management Int J Methods Psychiatr Res 2003 12 34 43 10.1002/mpr.140 12830308 PMC6878426

[B21] Hahn SR Thompson KS Wills TA The difficult doctor-patient relationship: somatization, personality and psychopathology J Clin Epidemiol 1994 47 647 57 10.1016/0895-4356(94)90212-7 7722577

[B22] Kroenke K Rosmalen JG Symptoms, syndromes, and the value of psychiatric diagnostics in patients who have functional somatic disorders Med Clin North Am 2006 90 603 26 10.1016/j.mcna.2006.04.003 16843765

[B23] Stanley IM Peters S Salmon P A primary care perspective on prevailing assumptions about persistent medically unexplained physical symptoms Int J Psychiatry Med 2002 32 125 40 10.2190/AVM3-8GU8-JW70-5RX5 12269594

[B24] Spitzer RL Kroenke K Williams JB Validation and utility of a self-report version of PRIME-MD: the PHQ primary care study. Primary Care Evaluation of Mental Disorders. Patient Health Questionnaire JAMA 1999 282 1737 44 10.1001/jama.282.18.1737 10568646

[B25] Mewes R Rief W Brahler E Lower decision threshold for doctor visits as a predictor of health care use in somatoform disorders and in the general population Gen Hosp Psychiatry 2008 30 349 55 10.1016/j.genhosppsych.2008.04.007 18585539

[B26] Härter M Kentgens M Brandes A Bock T Dirmaier J Erzberger M Fürstenberg W Hillebrandt B Karow A Knesebeck O König HH Löwe B Meyer HJ Romer G Rouhiainen T Scherer M Thomasius R Watzke B Wegscheider K Lambert M Rationale and content of psychenet: the Hamburg Network for Mental Health Eur Arch Psychiatry Clin Neurosci 2012 published online 13 September 2012 10.1007/s00406-012-0359-y 22972562

[B27] Inc P PHQ-Screeners 2002 http://www.phqscreeners.com. 19 March 2013

[B28] Löwe BSR Zipfel S Herzog W Gesundheitsfragebogen für Patienten (PHQ D). Komplettversion und Kurzform. Testmappe mit Manual, Fragebögen, Schablonen. 2. Auflage 2002 Karlsruhe: Pfizer

[B29] APA Diagnostic and Statistical Manual of Mental Disorders DSM-IV-TR (4th edition) 2000 Washington DC: American Psychiatric Press

[B30] Lowe B Kroenke K Herzog W Measuring depression outcome with a brief self-report instrument: sensitivity to change of the Patient Health Questionnaire (PHQ-9) J Affect Disord 2004 81 61 6 10.1016/S0165-0327(03)00198-8 15183601

[B31] Kroenke K Spitzer RL Williams JB The PHQ-9: validity of a brief depression severity measure J Gen Intern Med 2001 16 606 13 10.1046/j.1525-1497.2001.016009606.x 11556941 PMC1495268

[B32] Gandek B Ware JE Aaronson NK Cross-validation of item selection and scoring for the SF-12 Health Survey in nine countries: results from the IQOLA Project. International Quality of Life Assessment J Clin Epidemiol 1998 51 1171 8 10.1016/S0895-4356(98)00109-7 9817135

[B33] Schumacher J Schumacher J, Klaiberg A, Brähler E SWLS - Satisfaction With Life Scale Diagnostische Verfahren zu Lebensqualität und Wohlbefinden 2003 Göttingen: Hogrefe 305 309

[B34] Diener E Emmons RA Larsen RJ The Satisfaction With Life Scale J Pers Assess 1985 49 71 5 10.1207/s15327752jpa4901_13 16367493

[B35] Pavot W Diener E Review of the Satisfaction With Life Scale Psychol Assess 1993 5 164 72

[B36] Lowe B Wahl I Rose M A 4-item measure of depression and anxiety: validation and standardization of the Patient Health Questionnaire-4 (PHQ-4) in the general population J Affect Disord 2010 122 86 95 10.1016/j.jad.2009.06.019 19616305

[B37] Han C Pae CU Patkar AA Psychometric properties of the Patient Health Questionnaire-15 (PHQ-15) for measuring the somatic symptoms of psychiatric outpatients Psychosom 2009 50 580 5 10.1176/appi.psy.50.6.580 19996228

[B38] Rief W Pilger F Ihle D Immunological differences between patients with major depression and somatization syndrome Psychiatry Res 2001 105 165 74 10.1016/S0165-1781(01)00338-9 11814536

[B39] Rief W Auer C Is somatization a habituation disorder? Physiological reactivity in somatization syndrome Psychiatry Res 2001 101 63 74 10.1016/S0165-1781(00)00240-7 11223121

[B40] Interian A Allen LA Gara MA Somatic complaints in primary care: further examining the validity of the Patient Health Questionnaire (PHQ-15) Psychosom 2006 47 392 8 10.1176/appi.psy.47.5.392 16959927

[B41] van Ravesteijn H Wittkampf K Lucassen P Detecting somatoform disorders in primary care with the PHQ-15 Ann Fam Med 2009 7 232 8 10.1370/afm.985 19433840 PMC2682971

[B42] Smith RC Lein C Collins C Treating patients with medically unexplained symptoms in primary care J Gen Intern Med 2003 18 478 89 10.1046/j.1525-1497.2003.20815.x 12823656 PMC1494880

[B43] Jackson JL O'Malley PG Kroenke K Antidepressants and cognitive-behavioral therapy for symptom syndromes CNS Spectr 2006 11 212 22 16575378 10.1017/s1092852900014383

[B44] Kroenke K Efficacy of treatment for somatoform disorders: a review of randomized controlled trials Psychosom Med 2007 69 881 8 10.1097/PSY.0b013e31815b00c4 18040099

[B45] Kleinstauber M Witthoft M Hiller W Efficacy of short-term psychotherapy for multiple medically unexplained physical symptoms: a meta-analysis Clin Psychol Rev 2011 31 146 60 10.1016/j.cpr.2010.09.001 20920834

